# Synthesis of Non-Toxic Silica Particles Stabilized by Molecular Complex Oleic-Acid/Sodium Oleate

**DOI:** 10.3390/ijms17111936

**Published:** 2016-11-19

**Authors:** Catalin Ilie Spataru, Raluca Ianchis, Cristian Petcu, Cristina Lavinia Nistor, Violeta Purcar, Bogdan Trica, Sabina Georgiana Nitu, Raluca Somoghi, Elvira Alexandrescu, Florin Oancea, Dan Donescu

**Affiliations:** National Research & Development Institute for Chemistry and Petrochemistry—ICECHIM, Spl. Independentei 202, 6th District, Bucharest 060021, Romania; catalin.spataru@icechim-pd.ro (C.I.S.); raluca.ianchis@icechim-pd.ro (R.I.); cristian.petcu@icechim-pd.ro (C.P.); violeta.purcar@icechim-pd.ro (V.P.); trica.bogdan@gmail.com (B.T.); sabina.nitu@icechim-pd.ro (S.G.N.); raluca.somoghi@icechim-pd.ro (R.S.); elvira.alexandrescu@icechim-pd.ro (E.A.); florinoancea07@gmail.com (F.O.); ddonescu@chimfiz.icf.ro (D.D.)

**Keywords:** oleic acid, sodium silicate, mesoporous silica, octadecyltriethoxysilane

## Abstract

The present work is focused on the preparation of biocompatible silica particles from sodium silicate, stabilized by a vesicular system containing oleic acid (OLA) and its alkaline salt (OLANa). Silica nanoparticles were generated by the partial neutralization of oleic acid (OLA), with the sodium cation present in the aqueous solutions of sodium silicate. At the molar ratio OLA/Na^+^ = 2:1, the molar ratio (OLA/OLANa = 1:1) required to form vesicles, in which the carboxyl and carboxylate groups have equal concentrations, was achieved. In order to obtain hydrophobically modified silica particles, octadecyltriethoxysilane (ODTES) was added in a sodium silicate sol–gel mixture at different molar ratios. The interactions between the octadecyl groups from the modified silica and the oleyl chains from the OLA/OLANa stabilizing system were investigated via simultaneous thermogravimetry (TG) and differential scanning calorimetry (DSC) (TG-DSC) analyses.A significant decrease in vaporization enthalpy and an increase in amount of ODTES were observed. Additionally, that the hydrophobic interaction between OLA and ODTES has a strong impact on the hybrids’ final morphology and on their textural characteristics was revealed. The highest hydrodynamic average diameter and the most negative ζ potential were recorded for the hybrid in which the ODTES/sodium silicate molar ratio was 1:5. The obtained mesoporous silica particles, stabilized by the OLA/OLANa vesicular system, may find application as carriers for hydrophobic bioactive molecules.

## 1. Introduction

Recent concerns regarding Earth depollution have generated studies on aqueous dispersions of the vesicular systems consisting of fatty acids from plant renewable resources [1,2]. These vesicular systems (RCOO^−^HOOCR) are formed from the unionized fatty acid (RCOOH) and its corresponding alkaline salt (RCOO^−^) [1,2]. Due to their origin, these associated dispersed systems, in the form of bilayered vesicles, are biocompatible, biodegradable, and non-toxic and can be classified in the green chemistry field [3].

The formation of these bilayered vesicles was investigated through a simple technique: the titration of clear micellar solutions of alkaline salts with acids (usually inorganic acids such as HCl) [1,2,4,5,6,7,8]. The concentration of undissociated acid increases with acid quantity (RCOOH), and the initial transparent layout becomes opaque due to the formation of a vesicular supra-molecular structure [4,5,6,7,8]. The dispersion’s pH [4,5,6,7,8], the ^13^C Nuclear Magnetic Resonance (NMR) signals [7], and the X-ray diffraction peaks [8] are modified due to vesicle formation.

Previous experimental results have proven that the fatty acid vesicular systems are stabilized both by the hydrogen bonds of carboxyl–carboxylate groups and by the formation of hydrocarbon chain associations [1,2,6,7,8].

A very interesting phenomenon regarding aqueous vesicular dispersions of the fatty acids for further applications was that they can be formed at a temperature higher than the melting point of the bilayered hydrocarbon aggregates [1,2,6,7,8]. Most of the potential applications of these aqueous dispersions are focused on the formation of alkyl double layer [1,2,3,4,5,6,7,8], and the presence of the hydrophobic components allows for the solubilization of various hydrophobic molecules (e.g., some superior alcohols [9,10], bioactive substances, drugs, antifungal, and dyes [1,2,5,9,10]).

The encapsulation of bioactive compounds in fatty acids vesicles dispersed in water can solve an inconvenience derived from the aggregates structure. In contrast to the phospholipids vesicles, which have a great stability, the fatty acid vesicles have a high dynamic between the water solubilized monomers and their supra-molecular organizations. For this reason, the fatty acid vesicles have a flip-flop characteristic [10,11,12].

A suggestive example regarding the vesicle structure stabilization may be the radical cross-linking of the conjugated double bond from linoleic acid [13]. In the vesicles formed by the acid (R–COOH) and the alkaline salt (R–COO^−^) couple, the hydrocarbon chains adopt a structure in which double bonds are sidebyside, allowing for an easy cross-linking with ammonia persulfate at 80°C. If 5-fluorouracil issolubilized in the initial vesicles, after the radical cross-linking, the release rate of the small molecular compound will belower than in the case of the initial vesicles [13].

Another type of material recommended for the controlled release of bioactive compounds is represented by the mesoporous silica [14,15,16]. This material is biocompatible and bioresorbing [15,16]. The synthesis of such materials is generally based on sol–gel processes that are carried out in the presence of various surfactants. For future applications, ecologic surfactants that provide a “greener” synthesis route are recommended [17].

In a previously published review, the existence of mesoporous silica that can be obtained without any surfactant is highlighted [18]. Such silica was prepared through the sol–gel reactions between alkyl trialkoxysilanes with tetraalkoxysilanes [19]. Nanostructured hybrids, in which alkyl groups are forming bilayers, were generated from derivatives containing alkyl chains longer than 12 carbon atoms. Additionally, a bilayer organization of the octadecyl chains was emphasized by the silica alkylation with octadecyltrialkoxysilanes [20,21,22].

Data presented above allowed us to finda new experimental pathway for the synthesis of silica particles starting from sodium silicate in aqueous dispersions of oleic acid vesicles (OLA). Thus, in our previous paper [23], the preparation of mesoporous silica particles containing octadecyl functions was described. The originality of the results was derived from the fact that silica nanoparticles were obtained through the partial neutralization of OLA with a Na^+^ cation from sodium silicate aqueous solutions. At a molar ratio OLA/Na^+^ = 2:1, the composition required for the formation of vesicles (OLA/OLANa = 1:1), in which there are equal concentrations of carboxyl and carboxylate groups, was achieved. The neutralization of OLA led to a pH decrease in the sodium silicate initial solution and to the production of silica particles. Octadecyltrimethoxysilane (ODTMS) was added to these water-stable dispersions in order to generate hydrophobic silica particles with octadecyl groups (C_18_). The interactions between octadecyl groups from silica and oleyl groups from surfactant vesicles resulted in the transformation of the entire mixture into semi-opaque gels [23].

Therefore, in this paper, we continued to investigate the hybrid dispersions formed by OLA, partially neutralized with sodium silicate; however, this time, for the alkylation of silica particles, the biocompatible octadecyltriethoxysilane (ODTES) was used instead of ODTMS. Thus, ecological and biocompatible dispersions were obtained, with potential further applications for dispersing bioactive products. The as-synthesized systems, with various ODTES/sodium silicate molar ratios, were characterized in terms of particle size, ζ potential, and water interaction with the dispersed hybrids. After drying the samples, the thermal behavior, the melting temperature intervals of the hydrocarbon phase, the FT-IR spectra, the N_2_ adsorption–desorption isotherms, and the final morphology (TEM) were analyzed.

To our knowledge, the preparation of non-toxic silica particles through sodium silicate interaction with ODTES and stabilized by the oleic acid/sodium oleate complex (OLA/OLANa) is reported for the first time in the present paper. It is expected that the strong interaction between the octadecyl and oleyl chains will improve the affinity of the silica matrix for the fat-soluble bioactive compounds and will reduce their release rate from the newly formed gels.

## 2. Results and Discussion

Different grafting degrees of silica particles with octadecyl groups were achieved by using various amounts of ODTES, as shown in Table 1. During the synthesis of the aqueous dispersions, it was observed that the viscosity of the final mixtures increased with the amount of alkyl triethoxysilane, and their appearance changed from homogeneous fluid dispersions (Samples 1, 5, and 6) to homogeneous gels (Samples 2, 3, and 4).

**Figure 1:**
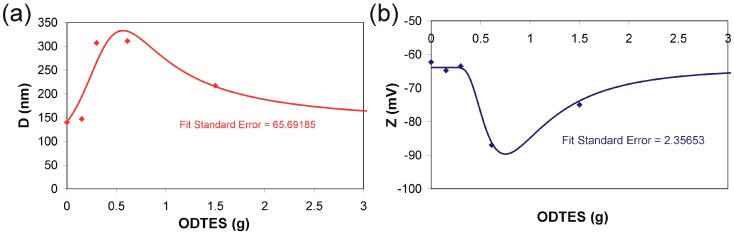
Evolution of (**a**) average diameter (D) and (**b**) ζ potential (Z) of the water dispersed particles, depending on the amount of octadecyltriethoxysilane (ODTES) added in the sol–gel system (The lines are for guiding the reader. The experimental data were plotted, and the given curve was fitted with Table Curve 2D version v5.01 software. The fit standard error calculated by the software is written inside each chart).

These observations suggest an intensification of the interactions between the octadecyl chains from the hydrolyzed ODTES and the oleyl groups from the OLA/OLANa vesicular complex. In order to demonstrate these interactions, the dispersions (diluted) were analyzed in regard to the average hydrodynamic diameter of the formed particles and their ζ potential (Figure 1a,b).

The variation in ODTES/silica particles dimensions is shown in Figure 1a. In good agreement with previous results, where alkylation was performed with ODTMS instead of ODTES [23], the maximum size was reached at a molar ratio ODTES/sodium silicate = 1:5. The same dispersion also has the highest negative value of ζ potential (Figure 1b). An explanation of these variable dependencies of the dimensions of the alkylated silica particles is the modification of the size and morphology of the aggregates arising from the association of elementary particles. By SAXS analyzes, a previously published study [22] revealed that, by increasing the ODTES concentration, the produced elementary particles change their morphology from fern-like structure to rough surface structure and to porous structure. These modifications have a great influence over the interaction of hybrid particles, which presents lamellar domains by the association of C_18_ chains with oleate chains associated as OLA/OLANa aggregates.

**Figure 2:**
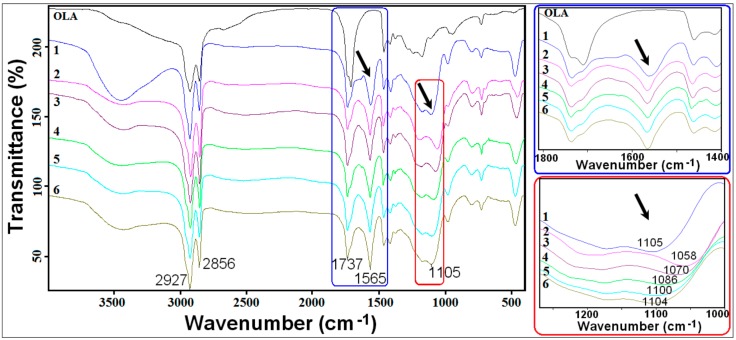
FT-IR spectra of ODTES/sodium silicate hybrids produced at different molar ratios. (The black arrows indicate the relevant peaks).

All FTIR spectra (Figure 2) of silica hybrids exhibit broad absorption bands in the 1000–1200 cm^−1^ range, which are assigned to the Si–O–Si stretching vibrations [24]. Two separate peaks are present, indicating two components from the Si–O–Si groups in the cyclic (~1170 cm^−1^) and linear (varying between 1050–1110 cm^−1^) structures. The cyclic structure of Si–O–Si (~1170 cm^−1^) is considered to be more condensed than the linear Si–O–Si [25]. For samples without ODTES or with a small amount of ODTES (Samples 1 and 6), the Si–O–Si peaks occur at about 1170 cm^−1^ (cyclic structure) and 1105 cm^−1^ (linear structure). With the increase in the amount of ODTES, the linear Si–O–Si peak is red-shifted and its intensity increases, indicating the presence of the hydrophobic functions of the silica hybrids prepared with ODTES.

Additionally, the asymmetric and symmetric stretching bands of CH_2_ groups around 2925 and 2857 cm^−1^ can be identified. The spectrum of pristine oleic acid shows a specific band at 1710 cm^−1^, caused by the C=O stretching vibration [26]. For the hybrid silica (Samples 1–6), this band is shifted to ~1736 cm^−1^, indicating a carboxylate interaction with a Na^+^ cation from sodium silicate. Moreover, a new band appears at ~1565 cm^−1^, assigned to COO^−^ stretching, proving the formation of the OLA/OLANa complex (the oleic acid and its sodium salt resulted from the interaction of the polar head-group of the oleate molecules with sodium cation) [26,27].

**Figure 3:**
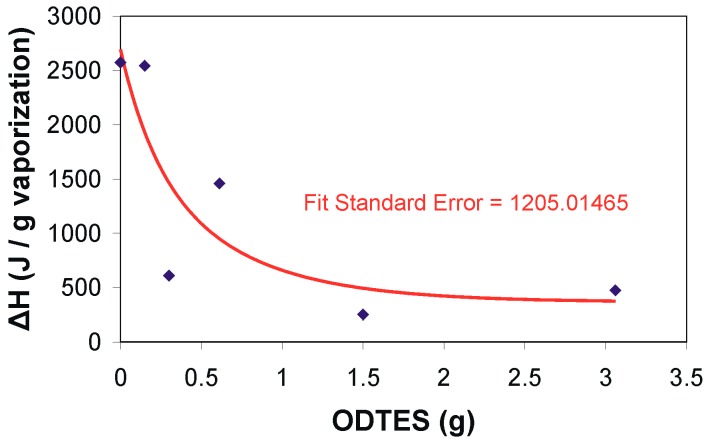
Variation in the vaporization enthalpy of the water dispersions as a function of the ODTES amount used for silica hydrophobization (The line is for guiding the reader. The experimental data were plotted, and the given curve was fitted with Table Curve 2D version v5.01 software. The fit standard error calculated by the software is written inside the chart).

The simultaneous thermogravimetry (TG) and differential scanning calorimetry (DSC) (TG-DSC) analysesallowed for the calculation of evaporation enthalpy assigned to the dispersion medium through programmed heating (Table 2, Figure 3). The evaporation of the dispersion medium occurred between 50 and 110 °C. A drastic decrease in the evaporation enthalpy and an increase in ODTES concentration in the final dispersions was observed (Figure 3). This behavior is attributed to the change in the dimension of the droplet dispersion of water in the hydrophobic medium. The water–water interaction is likely weaker than that of the water-hydrophobic region.

The decrease in water evaporation enthalpy from the silica dispersions with the increase in the degree of ODTES alkylation indicates a weak interaction of the water existing in the system with the molecular aggregates formed by the interaction of octadecyl and oleate chains. Our results are in good agreement with previously published data, where the decrease in water evaporation enthalpy along with the increase in the concentration of other hydrophobic groups is emphasized (e.g., amino acids [28], perflouorosulfonated membrane [29], polystyrene latexes [30], and polyvinyl alcohol [31]).

The existence of these interactions was also evidenced through an additional experiment. A supplementary dispersion (not shown here) in which the alkylation agent was aminopropyltriethoxysilane, in an equimolar ratio with sodium silicate, was synthesized through the same procedure described in the Materials and Methods section (similar to Sample 2). However, the evaporation enthalpy of the dispersion medium looked more like the enthalpy recorded for Sample 1 (containing silica particles obtained without ODTES) compared with that of Sample 2 (obtained with the highest amount of ODTES). This suggests that, in the absence of along hydrocarbon chain (C_18_), the occurrence of additional interaction of the resulted silica particles was no longer favored. Another observation was related to the aspect of the supplementary dispersion, which was a homogeneous fluid dispersion and not a gel like Sample 2.

The analysis of dried silica hybrids alkylated with C_18_ chains revealed their lamellar structuring [21]. Within the present paper, the interaction of the OLA/OLANa complex with such lamellar structures is explored for the dried synthesized hybrids through simultaneous TG-DSC analyses.

In our previous paper [23], it was shown that, in the dried hybrids prepared in the presence of olive oil (a triglyceride added in small quantities compared to oleic acid), the oleyl chains show a transition at ~15 °C. Additionally, the transition assigned to oleyl groups no longer occurred for hybrids prepared without olive oil. These results were the first proof that the solubilization of hydrophobic bioactive products occurs through interaction with the stabilization system (the OLA/OLANa complex).

To further investigate this phenomenon, in the present paper, the measurements were performed in a novel approach (Table 2). First, the dried silica hybrids were heated from room temperature up to +60 °C (1st heating run). Next, they were cooled down to −40 °C (1st cooling run), after which they were reheated up to +60 °C (2nd heating run). The obtained results are given in Table 2. It can be noticed that the oleyl groups alone do not present a specific transition—not even through this thermal treatment (Sample 1). At a molar ratio of ODTES/sodium silicate =1:1 (Sample 2), the hybrid presents a phase transition around 50 °C for all three heating/cooling runs. The enthalpy associated to this transition decreases with the concentration of the C_18_ groups, indicating that only the octadecyl chains are modified.

**Figure 4:**
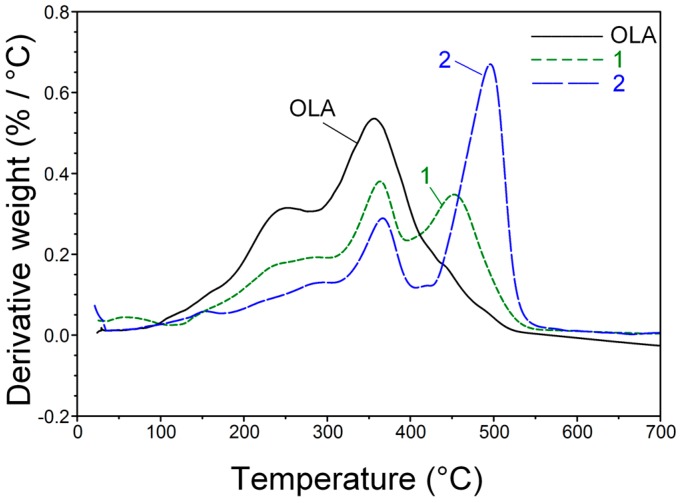
DTA diagrams for the pristine silica hybrid (Sample 1) and for ODTES-modified silica hybrid (Sample 2—ODTES/sodium silicate = 1 mol/1 mol).

To obtain new information regarding the hydrocarbon chains from the dried hybrids, thermal degradation was performed, and the weight loss (TGA) and the thermal effects that follow these degradations were simultaneously observed (DSC). The DTA curves of the pristine OLA and of Samples 1 and 2, shown in Figure 4, are useful to delimit the temperature domains in which thermo-destruction occurs.

The pristine OLA (Figure 4) exhibits a significant thermal degradation between 200–400 °C, with a maximum at 356 °C (T2). The hybrid prepared only with sodium silicate and stabilized by the OLA/OLANa complex (Sample 1) has a different behavior. It presented a weight loss at 364 °C (T2) and at 457 °C (T3). This second step of decomposition is due to a strong interaction of the stabilizer with the formed silica nanoparticles.

An important modification can be observed for the hybrid containing an equimolar ratio of ODTES and sodium silicate (Sample 2), in which the highest ODTES concentration among the studied hybrids can be found. In this case, a maximum decomposition rate occurs at ~336 °C (T2) and another at 495 °C (T3). This last thermal decomposition step is due mainly to the C_18_ chains decomposition and to the oleyl chains interacting with silica. This degradation occurred in a temperature range similar to the degradation recorded for the octadecyl-modified silica hybrids prepared without a stabilizing agent, in good agreement with previously published data [21].

**Figure 5:**
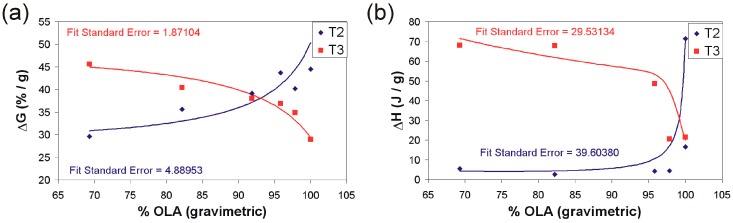
Evolution of (**a**) the weight loss in the 200–400 °C (T2) and respectively 400–700 °C (T3) temperature ranges and (**b**) the enthalpy corresponding to the all hybrids thermal decomposition steps (The lines are for guiding the reader. The experimental data were plotted, and the given curve was fitted with Table Curve 2D version v5.01 software. The fit standard error calculated by the software is written inside each chart).

Results represented in Figure 5 reveal a significant modification of OLA in the presence of silica. The weight losses, as well as the decomposition enthalpies, are different from those of the pure OLA. Variation in weight losses in the two temperature ranges 200–400 °C (T2) and 400–700 °C (T3) is represented in Figure 5a. Therein, an increase in weight losses for T2 together with an increase in gravimetric percentage of OLA (g OLA/g (OLA + C_18_) ×100) can be observed. Furthermore, a decrease in weight losses for T3 with an increase in the OLA fraction can also be observed.

The obvious difference between decomposition enthalpies of the hydrophobic components—the oleyl chains (T2) and the C_18_ + oleyl (T3)—demonstrates a more reduced thermal stability for the oleic acid chains that are not interacting with the silica nanoparticles. If the weight losses of the hybrids are recalculated considering the prevalence of the concentration of the oleyl chains, it can be estimated that ~50% of the OLA present in the hybrid film will be lost over the T2 interval. For this reason, the weight loss presented in Figure 5a increases in direct proportion with OLA content. For the T3 temperature interval, the above observation is no longer valid. The thermo-destruction of both the C_18_ chains and the oleyl chains attached to the silica particles occurs over this range (Figure 5a). In agreement with the weight losses, the enthalpies corresponding to the decomposition steps are modified with the composition of the hydrophobic phase (OLA + C_18_) (Figure 5b).

**Figure 6:**
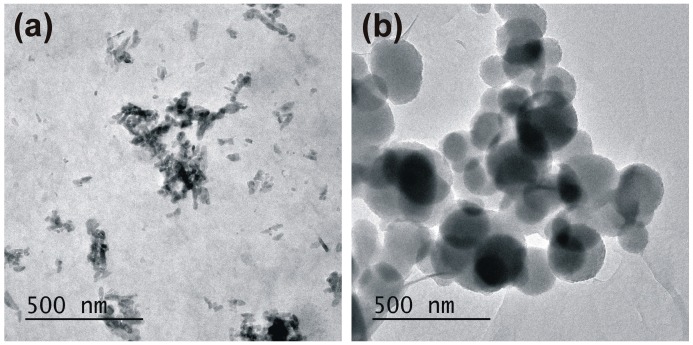
TEM images of (**a**) the octadecyl-modified silica particles witha 1:1 ODTES/sodium silicate molar ratio (Sample 2) and (**b**) the octadecyl-modified silica particles with an ODTES/sodium silicate molar ratio = 1:10 (Sample 5).

Figure 6 shows two representative TEM images recorded from silica particles after the removal by centrifugation of the stabilizing complex OLA/OLANa. The removal of the unbound organic molecules clearly enhances the quality of the TEM images of the silica particles. TEM images demonstrate that the size and morphology of the final particles is strongly dependent on the OLA-ODTES hydrophobic interactions. With the significantly increased dosage of ODTES (Sample 2), the formation of aggregates with lamellar morphology was confirmed by the TEM image shown in Figure 6a. For a lower amount of ODTES (Sample 5), it can be seen (Figure 6b) that the resulted silica particles are generally spherical, with a smooth surface and a polydisperse size distribution.

**Figure 7:**
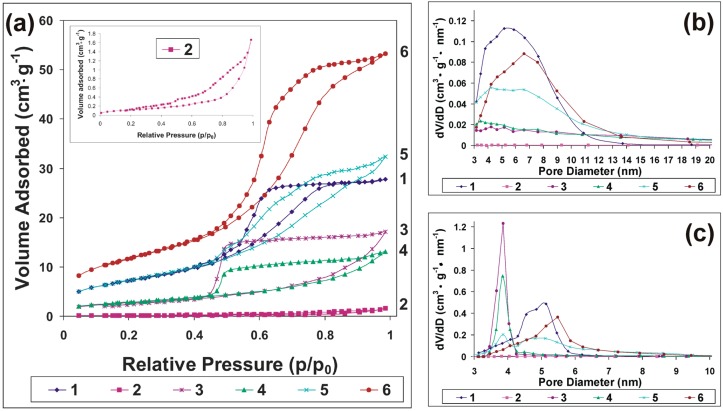
(**a**) N_2_ adsorption-desorption isotherms for the synthesized hybrids (Inset: enlarged diagram of N_2_ adsorption-desorption isotherm of Sample 2); (**b**) the corresponding BJH pore size distribution from the adsorption branch; (**c**) the corresponding BJH pore size distribution from the desorption branch.

The corresponding adsorption-desorption isotherms for the calcined silica hybrids are shown in Figure 7. All samples exhibit Type IV isotherms, proving that the synthesized silica hybrids stabilized by oleic acid are mesoporous. As shown, different hysteretic behaviors were recorded, depending on the silica precursor’s molar ratio.

Thus, for Sample 1 (without ODTES), a H2 hysteresis can be observed, indicating a classical pore blocking/percolation mechanism, due to constrictions resulting from ink-bottle pore effects. The H2 hysteresis loop is specific for materials with a disordered structure, and the pore size distribution and shape are not well defined. Sample 6 (containing the lowest amount of ODTES) also shows a H2 hysteresis loop. Hysteresis in the pore networks is more complex; very often, hysteresis loops that reflect the shapes of types H2 to H3 or to H4 are observed. Two basic mechanisms of desorption in the pore networks are distinguished as pore blocking/percolation and cavitation. For Sample 5 (ODTES/silicate = 1:10), a H2 to H3 hysteresis is still shown, but the cavitation mechanism becomes more involved in the desorption process. Isotherms with type H3 hysteresis do not exhibit any limiting adsorption at high *p*/*p*_0_. This behavior can, for instance, be caused by the existence of non-rigid aggregates of plate-like particles or assemblages of slit-shaped pores and basically should not be expected to provide a reliable assessment of either the pore size distribution or the total pore volume [32].

The desorption branches of Samples 3 and 4 exhibit a steep region associated with a forced enclosure of the hysteresis loop at ~0.47 *p*/*p*_0_. The shape of the H3 hysteresis loop shown by the two samples is associated with the delayed condensation and cavitation mechanism. It can be seen that, in contrast to the rest of the samples, the equimolar ODTES/silicate hybrid (Sample 2) yields a type III-like isotherm. However, a deeper look (inset of Figure 7a) reveals a negligible H3 hysteresis loop. The N_2_ adsorption isotherm at −197 °C of Sample 2 also shows the isotherm’s “forced closure” at the relative pressure (*p*/*p*_0_) of ~0.47.

The specific surface area calculated by the Brunauer–Emmett–Teller BET method (S_BET_) shows a significant decrease with the increase in the amount of ODTES (Table 3). A similar trend was confirmed by the Barrett–Joyner–Halenda (BJH) calculation of the specific area. Additionally, the total pore volume shows the same dependency with the ODTES amount.

The pore size distributions calculated from the adsorption branch (Figure 7b) revealed that the calcined silica hybrids are mesoporous, with most of the pores in the 2–15 nm range. The pore size distribution was calculated from the adsorption branch of the isotherm to avoid the tensile strength effect, which has a high impact on the results calculated from the desorption branch, as can be seen in Figure 7c.

Thus, for Sample 1, only mesopores and no macropores are formed. For Samples 5 and 6, the asymmetric region above 0.8 *p*/*p*_0_ reflects wide (secondary) mesopores with sizes >10 nm that do not necessarily have a significant volume [32]. Samples prepared with a high amount of ODTES shows very different size distribution curves for adsorption and desorption branches. For Sample 2, even if pore sizes of ~3.5 nm could be calculated by the BJH method from the adsorption branch, a much smaller surface area (~3 m^2^/g) and total pore volume (0.017 cm^3^/g) were measured, likely due to the incomplete calcination of the organic components of the hybrids.

## 3. Materials and Methods

### 3.1. Materials

Oleic acid (OLA) (Sigma-Aldrich, Steinheim, Germany), octadecyltriethoxysilane (ODTES 98%, Alfa Aesar, Karlsruhe, Germany), ethanol (S.C. Chimreactiv SRL, Bucharest, Romania), ammonium hydroxide 25% (S.C. Chimreactiv SRL), and sodium silicate (technical grade: 27.6% SiO_2_, 14.2% Na_2_O; S.C. Rasin SRL) were used as received.

### 3.2. Synthesis of Aqueous Dispersion

The aqueous dispersions were synthesized as in ref [23] through a method adapted from [20,21,22]. All the synthesized samples were run in duplicates. 4.2 g of OLA, 1.6 g of sodium silicate, and 40 mL of water were gradually added in a reactor provided with a magnetic stirrer. The whole mixture was kept under stirring (500 rpm) for 10 min at 40 °C. The resulted mixture generated a molar ratio OLA/Na = 2:1. In the same conditions of stirring and temperature, 3.07 g of ODTES (Sample 2, Table 1) solubilized in 8 mL of ethanol were added. The mixture was maintained at 40 °C for 4 h. The resulted aqueous dispersions were stored at room temperature for a minimum of 24 h in order to reach equilibrium. Next, one fraction was stored in sealed glass vials, and another fraction was poured in open polyethylene recipients and left to dry in air.

### 3.3. Characterization Methods

#### 3.3.1. Dynamic Light Scattering (DLS) and Laser Doppler Velocimetry (LDV)

Dynamic light scattering (DLS) and laser doppler velocimetry (LDV) techniques were used to examine particles size distribution and ζ potential, respectively, using a Zetasizer Nano ZS instrument (Malvern Instruments Ltd., Malvern, UK). An amount of 0.4 g of the obtained silica-OLA-ODTES hybrids were diluted in 25 mL of distilled water and ultrasonicated for 10 min at 50 °C. Samples were equilibrated for 10 min before being analyzed at 50 °C. The size distribution by intensity was considered for the evaluation of the hydrodynamic average diameter. Disposable DTS0012 polystyrene cells were used during the size experiments. The same dispersions prepared for size analyses were also subjected to ζ potential measurements using DTS 1060 disposable cells.

#### 3.3.2. Fourier Transformed Infrared (FTIR)

Fourier transformed infrared (FTIR) spectra were quantitatively (1‰) obtained in transmittance mode on KBr pallets, in the 400–4000 cm^−1^ spectral domain, using a spectrophotometer Tensor 37 (Bruker, Billerica, MA, USA).

#### 3.3.3. Simultaneous Thermal Analyses (STA)

Simultaneous thermal analyses (STA), which refers to the simultaneous application of Thermogravimetry (TG) and Differential Scanning Calorimetry (DSC) analyses to a single sample in a single instrument, were performed using a TA Instrument Q600 (TA Instruments, Lindon, UT, USA) at a 10 °C/min heating rate in an inert atmosphere (He respectively). Analyses were performed on both the as-synthesized aqueous dispersions (see Figure 3) as well on the dried hybrids (see Figure 4 and Figure 5).

#### 3.3.4. Transmission Electron Microscopy (TEM)

The morphologies of OLA-ODTES-silica hybrids were investigated by transmission electron microscopy (TEM), employing a Tecnai™ G2 F20 TWIN Cryo-TEM instrument (FEI Company, Hillsboro, OR, USA) at 200 kV acceleration voltages. The samples were observed directly without further staining to improve contrast. The excess of the OLA/OLANa stabilizing complex was washed and removed via centrifugation as described in the sample preparation before calcination (see N_2_ adsorption-desorption measurements). A droplet of diluted washed sample was poured on a carbon film coated copper grid and left to dry in air at room temperature.

#### 3.3.5. N_2_ Adsorption-Desorption

N_2_ adsorption-desorption measurements were carried out on a volumetric adsorption analyzer Quantachrome Nova2200e (Quantachrome Instruments, Boynton Beach, FL, USA) at liquid nitrogen temperature (−196 °C). Before calcination and textural characterization of the synthesized silica hybrids, the obtained aqueous dispersions were treated with ammonia excess to form ammonium oleate (OLANH_4_), soluble in water. This process was carried out with an addition of 50 mL of water that contained a sufficient quantity of ammonium hydroxide (the solution pH was ~9) to neutralize the excess of OLA. The excess of OLA was further washed and removed via centrifugation. The upper aqueous phase was removed. These operations were repeated twice. After water removal, the hybrids were dried and subsequently thermally treated as in [23] to obtain mesoporous silica.

## 4. Conclusions

The present paper describes an original method of obtaining non-toxic mesoporous silica, based on sodium silicate and oleic acid. Due to the interactions between the octadecyl groups from the modified silica and the oleyl chains from the OLA/OLANa stabilizing system, the reaction mixtures were converted to stable semi-opaque gels.

The silica functionalization with ODTES was performed at different ODTES/Na silicate molar ratios. FTIR spectra revealed the occurrence of the OLA/OLANa complex and the influence of the ODTES amount on the silica network formation. Simultaneous TG-DSC analysis allowed for the measurement of the heat flow variation with the temperature, as well as the calculation of the water’s enthalpy of vaporization for the resulted silica latexes. Through thermal analysis, the increase in weight losses due to the higher percentage of hydrophobic components present in the dried hybrids was highlighted. DSC analyses revealed a modification of the water evaporation enthalpy caused by an increase in the silica hydrophobicity.

In good agreement with previously published data, the DLS measurements and TEM images work to distinguish the effect of the hydrophobic interaction between OLA and ODTES on the hybrid’s final morphology. The N_2_ adsorption measurements show that the obtained hybrids are mesoporous. The increase in the degree of octadecyl substitution led to significant modifications of the pores’ shape, dimension, and size distribution.

The obtained non-toxic silica particles may be used as carriers for various hydrophobic bio-active substances.

## Figures and Tables

**Table 1:** Compositions of the investigated silica hybrids.

Sample	ODTES (g)	ODTES/Sodium Silicate (mol/mol)	OLA/OLA + ODTES (mol/mol)	D ^a^ (nm)	Z ^b^ (mV)	Appearance
1	0	Onlysodium silicate	1	140 ± 1.124	−63.3 ± 1.35	opaque fluid
2	3.07	1:1	0.67	163 ± 0.845	−67.8 ± 1.53	opaque gel
3	1.5	1:2	0.805	217 ± 1.567	−60.5 ± 0.948	opaque gel
4	0.61	1:5	0.91	311 ± 1.499	−87.2 ± 2.21	opaque gel
5	0.3	1:10	0.954	307 ± 3.303	−63.0 ± 2.56	opaque fluid
6	0.15	1:20	0.968	147 ± 1.130	−64.8 ± 1.73	opaque fluid

^a^ = hydrodynamic average diameter of the synthesized silica particles; ^b^ = ζ potential.

**Table 2:** Thermogravimetry (TG) and differential scanning calorimetry (DSC) (TG-DSC) coupled analysis results for the octadecyl-modified silica hybrids.

Sample No.	Water Dispersions	Dried Hybrids					
	DSC	TGA/DTG (ΔG%/T_max_ °C)				DSC (ΔH (J/g)/T_i_ (°C))	
	10–200 °C	0–200 °C	200–400 °C	400–700 °C	Residue at 700 °C %	200–400 °C	400–700 °C
	ΔH (J/g)	ΔG	ΔG/T_max_ 2	ΔG/T_max_ 3		ΔH/T_i_2	ΔH/T_i_3
1	2573	8.69	44.50:364.0	28.94:457.0	17.88	16.49:337	21.45:426
2	472	6.59	29.63:366.7	45.64:495.0	18.25	5.56:338	68.1:466
3	249	5.46	35.60:369.1	40.43:488.6	18.51	2.63:385	67.92:448.3
4	1458	6.94	39.14:367.1	38.99:471.7	14.96	5.29:322.9	12.28:480.3
5	608	7.14	43.70:367.8	36.92:470.7	12.25	4.22:382.2	48.7:454.7
6	2543	6.48	40.15:363.8	34.90:488.6	18.51	4.43:375.3	20.82:452.5
OLA	–	10	73.6:356	16.3:–	0	71.3:370	–

**Table 3:** Textural properties of the C18-modified silica hybrids from N_2_ adsorption-desorption isotherms.

Sample	S_BET_ (m^2^·g^−1^)	S_BJH_ _ads._ (m^2^·g^−1^)	D_a_^a^ (nm)	D_d_^b^ (nm)	Pore Volume ^c^ (m^3^·g^−1^)	V_t_^d^ (m^3^·g^−1^)
1	380.202	416.509	5.19	5.14	0.621	0.624
2	2.868	3.560	3.54	4.05	0.017	0.0167
3	114.364	104.409	4.22	3.86	0.308	0.318
4	165.261	115.717	3.47	3.86	0.293	0.325
5	291.300	268.893	4.20	3.86	0.524	0.551
6	294.393	319.468	6.60	5.49	0.572	0.571

^a^ = Pore diameter (BJH adsorption branch); ^b^ = Pore diameter (BJH desorption branch); ^c^ = Pore volume (BJH adsorption branch); ^d^ = Total pore volume is estimated to be the liquid volume of nitrogen at *p*/*p*_0_ = 0.98.
